# A New Feature Extraction Method Based on Improved Variational Mode Decomposition, Normalized Maximal Information Coefficient and Permutation Entropy for Ship-Radiated Noise

**DOI:** 10.3390/e22060620

**Published:** 2020-06-03

**Authors:** Dongri Xie, Haixin Sun, Jie Qi

**Affiliations:** 1 School of Electronic Science and Engineering, Xiamen University, Xiamen 361005, China; 23320171153152@stu.xmu.edu.cn; 2 School of Informatics, Xiamen University, Xiamen 316005, China; hxsun@xmu.edu.cn

**Keywords:** ship-radiated noise, feature extraction, improved variational mode decomposition, maximal information coefficient, permutation entropy

## Abstract

Due to the existence of marine environmental noise, coupled with the instability of underwater acoustic channel, ship-radiated noise (SRN) signals detected by sensors tend to suffer noise pollution as well as distortion caused by the transmission medium, making the denoising of the raw detected signals the new focus in the field of underwater acoustic target recognition. In view of this, this paper presents a novel hybrid feature extraction scheme integrating improved variational mode decomposition (IVMD), normalized maximal information coefficient (norMIC) and permutation entropy (PE) for SRN signals. Firstly, the IVMD method is employed to decompose the SRN signals into a number of finite intrinsic mode functions (IMFs). The noise IMFs are then filtered out by a denoising method before PE extraction. Next, the MIC between each retained IMF and the raw SRN signal and PE of retained IMFs are calculated, respectively. After this, the norMICs are used to weigh the PE values of the retained IMFs and the sum of the weighted PE results is regarded as the classification parameter. Finally, the feature vectors are fed into the particle swarm optimization-based support vector machine multi-class classifier (PSO-SVM) to identify different types of SRN samples. The experimental results have indicated that the classification accuracy of the proposed method is as high as 99.1667%, which is much higher than that of other currently existing methods. Hence, the method proposed in this paper is more suitable for feature extraction of SRN signals in practical application.

## 1. Introduction

Signal analysis of ship-radiated noise (SRN) is important, not only for military operations but also for environmental protection [1,2]. Feature extraction of SRN signals can improve the performance of detection equipment to some extent [3,4]. However, the presence of various types of marine environmental noise significantly increase the difficulty of extracting features that reflect the intrinsic characteristics of the ships [5,6]. In recent years, an increasing number of studies have been focused on the feature extraction of SRN signals with satisfactory results. Unfortunately, these approaches inevitably introduce additional problems. For example, the Fourier transform (FT) [7] is only valid in providing the spectral information of the signal, but fails to work on its time-varying characteristics. To address this issue, short-time Fourier transform (STFT) is proposed to exhibit the time-varying information of the analyzed signal. Nevertheless, owing to the window function with fixed length, STFT is doomed to be incapable of balancing the time-domain and frequency-domain resolution of the observed signals [8]. For the wavelet transform (WT) [9], the performance of time-frequency analysis is improved, but the wavelet basis function and decomposition layers need to be set in advance, which virtually impedes its further application in practice [10].

Previous studies have already demonstrated the non-linear properties of SRN [11,12]. As one of the most important basic theories of thermodynamics, entropy is a suitable candidate to address the complex metrics of time series. Entropy can accurately reflect the dynamic changes of nonlinear dynamic systems. The entropy value changes with the dynamic change of the time series. A larger entropy value means higher complexity of the time series; while a smaller entropy value means lower complexity of the time series, and vice versa. Up to now, there have been emerging entropy algorithms to quantify the uncertainty of time sequence, such as approximate entropy (AE) [13], sample entropy (SE) [14] and permutation entropy (PE) [15]. As PE enjoys the merits of being conceptually simple and computationally fast, since proposed, it has been widely used in the fields of EEG signal research [16], financial sequence analysis [17] and mechanical fault diagnosis [18]. A study has proven the better stability of PE than that of AE and SE [19], but PE only compares the neighboring values without consideration of amplitude, meanwhile, it is also greatly influenced by the equal value, which results in a deviation between the calculated value and the true value. To address this issue, the weighted permutation entropy (WPE) was proposed in [20], it considers the fluctuation of the signal with the weighting factor introduced. Compared with PE, WPE is more sensitive to amplitude information, which can effectively detect the stagnation area and abrupt area of the signal. As an enhanced method of PE, WPE has gradually attracted the attention of scholars and has been practically applied in many fields [21,22,23]. A new kind of permutation entropy (RPE) was put forward by Bandt to analyze EEG signals in [24]. RPE measures the distance between the observed signal and the Gaussian white noise, showing the opposite trend to the traditional entropy algorithms such as AE, SE, PE and WPE. In addition, RPE exhibits strong stability in terms of data length. Inspired by RPE, the reverse weighted permutation entropy (RWPE) integrates amplitude and distance information, which has stronger noise recognition ability than PE and RPE, as well as better stability than PE, RPE and WPE [25].

Despite the importance of entropy methods, they suffer in the presence of marine environmental noise. One way to suppress the marine environmental noise is through signal decomposition algorithms. A large number of signal decomposition methods have emerged to further improve the performance of feature extraction, such as empirical mode decomposition (EMD) [26], ensemble empirical mode decomposition (EEMD) [27], complementary EEMD (CEEMD) [28],variational mode decomposition (VMD) [29], etc. Each of these algorithms enjoys its own unique merits, but also reveals defects. As a parameter-free method, EMD decomposes the signal into a set of finite intrinsic mode functions (IMFs), the core of which is the Hilbert transform (HT). The purpose of HT is to obtain the instantaneous frequency of the component. Therefore, EMD can accurately express and describe the instantaneous frequency of different components, which is suitable for analysis of nonlinear and non-stationary signals. Study in [30] combined EMD and matrix factorization for blind source separation of speech signals. Experimental results have proved its advantages over other methods. However, the main drawback of the EMD method is mode mixing and many researchers have been tried hard to address this issue. EEMD has been presented as an improved version of EMD. It has solved the issue of mode mixing parasitism in EMD by adding Gaussian white noise to the analyzed signal, and finally averaging the results of multiple decompositions to obtain the IMFs. EEMD, however, poses additional challenges. Firstly, as with EMD, in the decomposition process, the residual components will also be decomposed by EEMD. Secondly, the randomness of Gaussian white noise inevitably results in the difference between each decomposition time, and residual noise is inevitably introduced. Also, in EEMD theory, there is a lack of supporting rigorous mathematical foundations. CEEMD attempted to remove the residual noise introduced by EEMD by adding pairs of positive and negative Gaussian white noise, but the number of IMFs between each decomposition result remains inconsistent. By contrast, variational mode decomposition (VMD) addressed the mode aliasing issue as well as avoided the introduction of residual noise. The VMD method assumes that each mode is around a center frequency with limited bandwidth. Therefore, in order to find the center frequency and bandwidth of each IMF, VMD continuously searches for each mode and its center frequency by using the alternating direction method of multiplier, thereby solving the variational problem. But the inherent weakness of VMD lies in the preset of input parameters, such as the mode number and quadratic penalty term.

A denoising processing always includes removal of noise IMFs, reconstruction of signal-dominant IMFs, or selection of one or more sensitive IMFs. In recent years, considerable denoising techniques combining signal decomposition and entropy methods have been continuously emerging with satisfactory results. Study in [31], a group of IMFs were first obtained from VMD decomposition of SRN signals, The IMF with the closest fluctuation-based dispersion entropy (FDE) value to the original signal is used as the sensitive IMF, realizing a classification accuracy of 97.5%. In a previous study [32], the improved complementary ensemble empirical mode decomposition with adaptive noise (ICEEMDAN) were first employed to decompose the SRN signals to calculate the IMFs. Removal of the noise-dominant IMFs was then completed using the mutual information (MI) between the obtained IMF and the raw signal. The multi-scale improved permutation entropy (MIPE) of each retained IMF weighted by the normalized MI finally served as the input of classifier, which has achieved higher recognition rate for SRN samples under different signal to noise ratio (SNR). Also, in [33], denoising processing was realized using the pure IMFs and the noise-dominant IMFs obtained based on DE and interval thresholding to reconstruct the new signal. The use of chaotic systems proved that this method has a higher SNR than other methods. Similarly, another denoising method [34] first used VMD to obtain a set of IMFs from SRN signal. The noise IMFs were then screen out according to a RPE threshold. Finally, the remaining components are used to reconstruct the signal. The feasibility and effectiveness of this method have been verified by simulation signals and measured signals. Despite the great achievements of these methods, flaws still exist in themselves. First of all, both [31] VMD-SMIF-FDE and [34] VMD-RPE took the number of IMFs by EMD as the reference of the mode number of VMD, in this case, the results of VMD decomposition remain questionably unreliable. In addition, the mode number of VMD were only highlighted by these two methods without consideration of other parameters, such as the quadratic penalty term, which was also the case in [19].

On this basis, this paper proposes a new feature extraction method for SRN signals using improved VMD (IVMD), normalized maximal information coefficient (norMIC), and PE. For the calculation of parameters in IVMD method, the range of mode number *K* is set to 5–15, and the quadratic penalty term α is set as 500–10000 with step size of 500, respectively. After this, the variance of the IMFs’ center frequency is calculated after each decomposition. The combination of *K* and α maximizing the variance is regarded as the optimal input parameters for IVMD. Then, a group of IMFs can be extracted by IVMD decomposition of SRN signals. The noise IMFs are then filtered out by a denoising method and the remaining ones are used for the subsequent feature extraction. After that, the MIC between each retained IMF and the raw SRN signal and PE of the retained IMFs are calculated, respectively. The MICs are employed to weigh the calculated PE values and the sum of the weighted PE is used as the recognition parameter of classifier.

IVMD overcomes the blindness of parameter selection in VMD such that it is employed to process SRN signals in this paper. As a pointer to correlation, MIC is first introduced to measure underwater acoustic signals. RWPE is a newly proposed uncertainty metric for time series, and it is also utilized for the first time to denoising of noisy signals. The scheme proposed in this paper comprehensively covers the steps of underwater acoustic target recognition, including signal decomposition, noise reduction, feature extraction and classification, which can serve as a supplement to the field of underwater acoustic signal processing, and also provide a reference for the basis of further research.

The structure of the paper is organized as follows: Section 2 presents the background. The basic steps of the proposed method are briefly introduced in Section 3. The proposed method is used for analysis of simulation signals in Section 4. In Section 5, the new technique is utilized for the test on measured data. Finally, the conclusions of the full text are drawn in Section 6.

## 2. Background

### 2.1. Variational Mode Decomposition

As an adaptive signal decomposition algorithm, variational mode decomposition (VMD) [29] can decompose the analyzed signal into a set of pseudo-orthogonal components, it defines the IMF as an amplitude-modulated-frequency-modulated (AM-FM) signal with respect to instantaneous frequency *Ph_k_*(*t*) and instantaneous amplitude *Am_k_* (*t*) given as:

(1) $$I_{k}\left(t\right)=Am_{k}\left(t\right)\cos\left(Ph_{k}\left(t\right)\right)$$

In the VMD algorithm, given the target signal *s*(*t*), partial derivative function $\partial_{t}$, and unit impulse function $\delta_{t}$, in order to search for each mode *I_k_*(*t*) and its center frequency *f_k_*. The optimization problem can be expressed as follows:

(2) $$\begin{array}{c} \min_{\left\{I_{k},f_{k}\right\}}\left\{{\sum_{k}\left\|\partial_{t}\left[\left(\delta \left(t\right)+\frac{j}{\pi t}\right)*I_{k}\left(t\right)\right]e^{-j2\pi f_{k}t}\right\|}_{2}^{2}\right\}\\ s.t.\sum_{k}I_{k}=s(t) \end{array}$$

The constraint problem in (2) can be converted into a new form by using Lagrange multipliers: λ:

(3) $$\begin{array}{l} L\left(\left\{I_{k}\right\},\left\{f_{k}\right\},\lambda \right)=\alpha {\sum_{k}\left\|\partial_{t}\left[\left(\partial_{t}+\frac{j}{\pi t}\right)*I_{k}\left(t\right)\right]e^{-j2\pi f_{k}t}\right\|}_{2}^{2}\\ +{\left\|s(t)-\sum_{k}I_{k}(t)\right\|}_{2}^{2}+\langle \lambda (t),s(t)-\sum_{k}I_{k}(t)\rangle \end{array}$$

where α denotes the penalty factor. $I_{k}^{n+1}$, $f_{k}^{n+1}$ and $\lambda^{n+1}$ can be updated as below:

(4) $${\hat{I}}_{k}^{n+1}(f)=\frac{\hat{s}(f)-\sum_{i\neq k}{\hat{I}}_{i}(f)+\frac{\hat{\lambda }(f)}{2}}{1+2\alpha {\left(f-f_{k}\right)}^{2}}$$

(5) $$f_{k}^{n+1}=\frac{\int_{0}^{\infty }2\pi f{\left|{\hat{I}}_{k}(f)\right|}^{2}df}{\int_{0}^{\infty }{\left|{\hat{I}}_{k}(f)\right|}^{2}df}$$

(6) $${\hat{\lambda }}^{n+1}(f)={\hat{\lambda }}^{n}(f)+\varepsilon (\hat{s}(f)-\sum_{k}{\hat{I}}_{k}^{n+1}(f))$$

where *ɛ* is the updated parameter. The VMD stop condition is given by:

(7) $${\sum_{k}\left\|{\hat{I}}_{k}^{n+1}-{\hat{I}}_{k}^{n}\right\|}_{2}^{2}/{\left\|{\hat{I}}_{k}^{n}\right\|}_{2}^{2}<a$$

where *a* represents the convergence accuracy.

### 2.2. Permutation Entropy

Since permutation entropy (PE) [15] only compares the neighboring values without consideration of the amplitude, it enjoys the merits of being conceptually simple and computationally fast. The brief calculation steps of PE are described below:

Given the embedding dimension *m* and the time delay τ, the time sequences $\left\{x\left(1\right),x\left(2\right),\cdots ,x\left(N\right)\right\}$ can be mapped to:

(8) $$X_{i}=\left\{x(i),x(i+\tau ),\ldots ,x(i+(m-1)\tau )\right\},i=1,2,\ldots ,N-\left(m-1\right)\tau$$

Vector *X_i_* is rearranged in an increasing order as:

(9) $$x(i+(j_{1}-1)\tau )\leq x(i+(j_{2}-1)\tau )\leq \cdots \leq x(i+(j_{m}-1)\tau )$$

and if two elements in *X_i_* are equal, like:

(10) $$x(i+(j_{1}-1)\tau )=x(i+(j_{2}-1)\tau )$$

then their order can be denoted as:

(11) $$x(i+(j_{1}-1)\tau )\leq x(i+(j_{2}-1)\tau )(j_{1}\leq j_{2})$$

In this case, a symbol sequences can be obtained as:

(12) $$S(g)=(j_{1},j_{2},\cdots ,j_{m})$$

*m*! symbol sequences can be calculated in total, where *S*(*g*) is one symbol sequences in phase space, g=1,2,…,k,k≤m!.

The probability distribution of all the symbol sequences is calculated as $P_{1},P_{2},\ldots ,P_{k}$, for convergence, the PE is defined as below:

(13) $$H_{p}(m)=-{\left(\ln m!\right)}^{-1}\sum_{g=1}^{k}P_{g}\ln P_{g}$$

The PE value ranges from 0 to 1. A larger *H_p_* means higher complexity of the time series; a smaller *H_p_* means lower uncertainty of the time series.

### 2.3. Reverse Weighted Permutation Entropy

The main defect of PE lies in the neglect of amplitude with only consideration of neighboring values. The weighted permutation entropy (WPE) proposed in [20] has addressed the PE issue. Reverse weighted permutation entropy (RWPE) fused distance and amplitude information, has shown a strong recognition ability of noise [25]. In the RWPE, for a given embedding dimension *m* and time delay τ, the weight $w_{j}$ of the embedding vector *X_i_* can be denoted as:

(14) $$w_{j}=\frac{1}{m}{\sum_{k=1}^{m}\left[x_{j+\left(k-1\right)\tau }-{\bar{X}}_{j}^{m,\tau }\right]}^{2}$$

(15) $${\bar{X}}_{j}^{m,\tau }=\frac{1}{m}\sum_{k=1}^{m}x_{j+\left(k+1\right)\tau }$$

The weighted relative frequency can be calculated as:

(16) $$p_{w}\left(\pi_{i}^{m,\tau }\right)=\frac{\sum j\leq N^{1_{u:\mathrm{type}\left(u\right)=\pi_{i}}\left({\bar{X}}_{j}^{m,\tau }\right)w_{j}}}{\sum j\leq N^{1_{u:\mathrm{type}\left(u\right)\in \Pi }\left({\bar{X}}_{j}^{m,\tau }\right)w_{j}}}$$

RWPE measures the distance between the observed signal and the Gaussian white noise, in which case the RWPE is defined as follows:

(17) $$H_{RWPE}\left(m,\tau \right)={\sum_{i:\pi_{i}^{m,\tau }\in \Pi }\left(p_{w}\left(\pi_{i}^{m,\tau }\right)-\frac{1}{m!}\right)}^{2}={\sum_{i:\pi_{i}^{m,\tau }\in \Pi }\left(p_{w}\left(\pi_{i}^{m,\tau }\right)\right)}^{2}-\frac{1}{m!}$$

### 2.4. Maximal Information Coefficient and Normalized Maximal Information Coefficient

Maximal information coefficient (MIC) [35] is a good candidate for measuring the correlation of paired variables. It is reliable and competent in reflecting the linear or nonlinear relationship between variables, and has higher accuracy than mutual information (MI). The calculation steps of MIC for paired variables X and Y can be briefly summarized as follows:

Step 1: The set of data points with two attributes is distributed in two-dimensional space. m×n grids are used to divide the data space, so that the frequency of data points in the (x, y) grid is taken as the estimation of p (x, y), let the frequency of the data point falling on the xth row be the estimate of p (x), and similarly obtain the estimate of p (y), namely:

(18) $$p\left(x,y\right)=\frac{N\left(x,y\right)}{N_{total}}$$

(19) $$p\left(x\right)=\frac{N\left(x\right)}{N_{total}}$$

(20) $$p\left(y\right)=\frac{N\left(y\right)}{N_{total}}$$

where $N\left(x,y\right)$ is the number of data points in the (x, y) grid, $N\left(x\right)$ is the number of data points falling on the xth row, $N\left(y\right)$ is the number of data points falling on the yth column, and $N_{total}$ is the total data points.

Step 2: Calculate the MI of random variables X and Y. As there are more than one way to divide the data points by a grid of m×n, we need to calculate a grid maximizing the MI. Then the normalization factor is used to convert the value of MI into the interval (0,1). Finally, find the grid resolution maximizing the normalized MI as a measure of MIC. By this way, the MIC is finally defined as follows:

(21) $$MIC\left(X,Y\right)=\underset{m\times n<B}{\max}\left(\frac{p\left(x,y\right)\log\frac{p(x,y)}{p(x)\cdot p(y)}}{\log\;\min\left\{m,n\right\}}\right)$$

where B is generally set to 0.6 power of the data size.

The noise IMFs by IVMD will be removed after a denoising processing. In order to weigh the PE values of the retained IMFs, the normalized MIC (norMIC) corresponding to each retained IMF is calculated as:

(22) $$norMIC_{i}=\frac{MIC_{IMF_{i},\;S}}{\sum_{j=1}^{k}MIC_{IMF_{j},\;S}},\;k<K$$

where *k* denotes the number of retained IMFs, *K* denotes the mode number of IVMD, and *S* is the raw SRN signal.

## 3. The Proposed Feature Extraction Method for Ship-Radiated Noise

**Figure 1:**
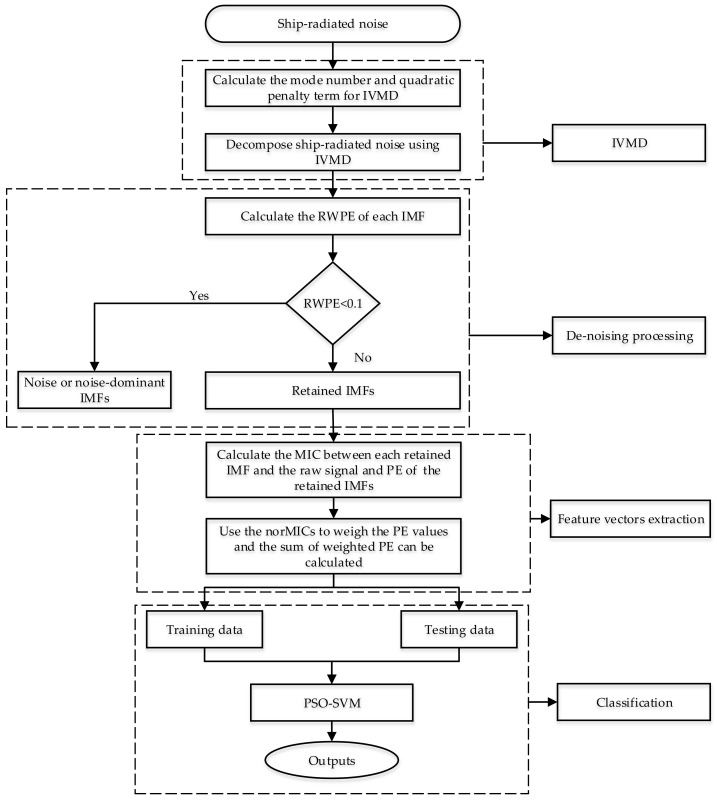
The flow chart of the proposed technique.

This paper proposes a hybrid feature extraction method for SRN signals integrating IVMD, norMIC and PE. The flow chart of the proposed method is presented in Figure 1. The main steps of the proposed method are as follows:

Step 1: The mode number *K* is set to 5–15, and the quadratic penalty term α is set as 500–10000 with step size of 500. Perform VMD method with combination of $\left(K_{i},\alpha_{j}\right)$, 1≤i≤length(K), 1≤j≤length(α), and calculate the variance of the IMFs’ center frequency after each decomposition.

Step 2: The combination of $\left(K_{optimal},\alpha_{optimal}\right)$ maximizing the variance is considered as the optimal parameters for IVMD.

Step 3: Decompose SRN signals using IVMD and a set of IMFs can be extracted.

Step 4: Extract the RWPE for each IMF and the sensitive IMFs are retained (RWPE≥0.1).

Step 5: Calculate the MIC between each retained IMF and the raw SRN signal and PE of retained IMFs, respectively.

Step 6: The MICs are employed to weigh the calculated PE values and the sum of the weighted PE is regarded as the recognition parameter of classifier. Namely, $SWPE=\sum_{i=1}^{k}norMIC_{i}\cdot PE_{i}$, k<K.

Step 7: The obtained feature vectors are randomly divided into the training data to train the classifier, and the remaining ones are the testing set for recognition.

Step 8: Finally, the test set is input to the classifier to realize the classification and recognition of underwater targets.

## 4. Simulation Signals Analysis

### 4.1. IVMD of Simulation Signals

Since SRN signals contain rich line spectral components, composite simulation signals composed of single frequency components are thus constructed to test the performance of IVMD. The simulation signals used in this paper are as follows:

(23) $$\left\{\begin{array}{l} f_{1}(t)=\cos(10\pi t)\\ f_{2}(t)=\cos(100\pi t)\\ f_{3}(t)=\cos(200\pi t)\\ f(t)=f_{1}(t)+f_{2}(t)+f_{3}(t)+\eta \end{array}\right..$$

where the data length and sampling frequency are set to 5000 and 1 kHz, respectively. η is 0.5 times the standard Gaussian white noise.

**Figure 2:**
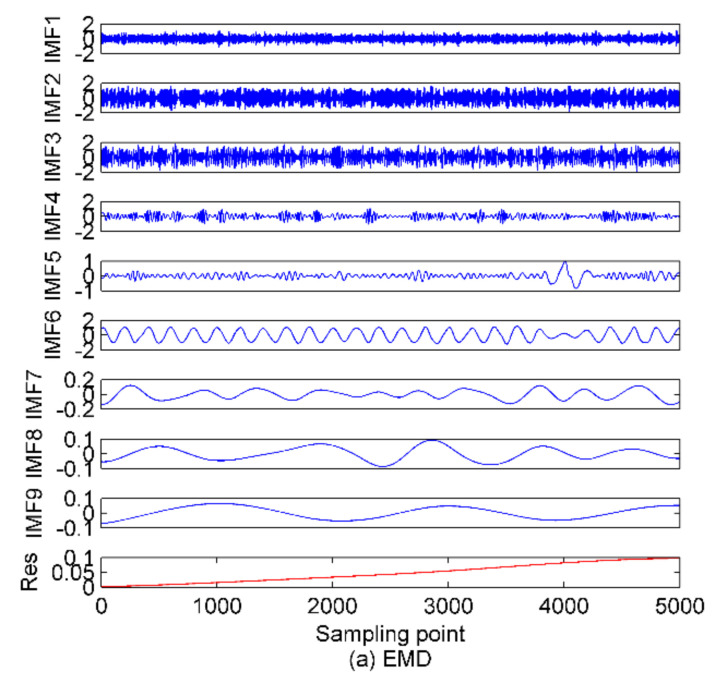

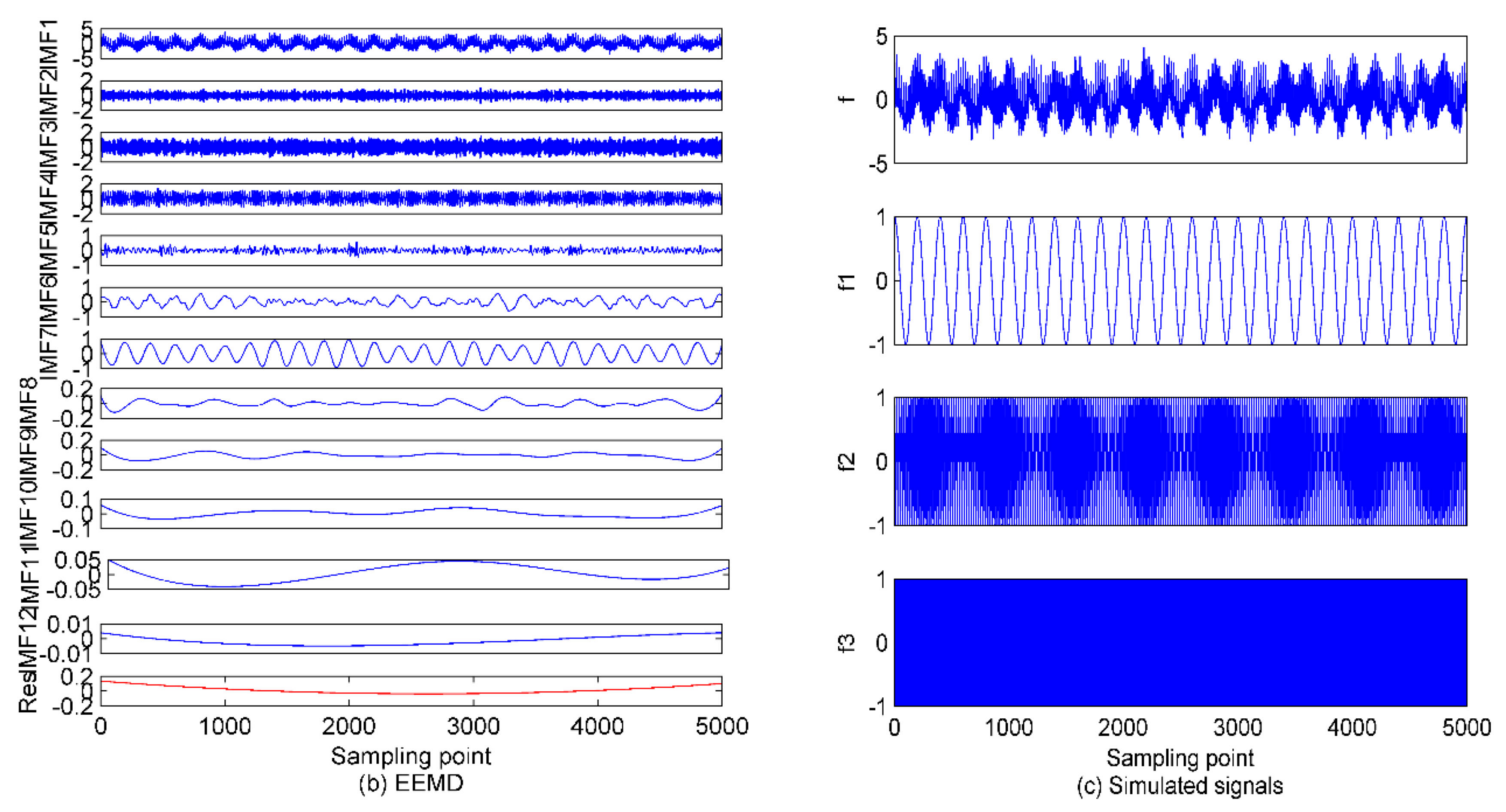
The decomposition results of EMD and EEMD and time-domain waveforms of simulation signals; (**a**) EMD method; (**b**) EEMD method; (**c**) the simulation signals.

As in [29], a properly selected *K* value greatly affects the decomposition result of VMD. A too large *K* will cause undesirable spurious components to be generated; a too small *K* will make the components with important information discarded in the decomposition process. Study in [19] only focused on the mode number of VMD without focus on other relevant parameters. In this paper, we also want to further explore the influence of quadratic penalty term α on the decomposition results. The EMD and EEMD methods are first used to process the simulation signals. The results and time-domain waveforms of the simulation signals are given in Figure 2.

**Figure 3:**
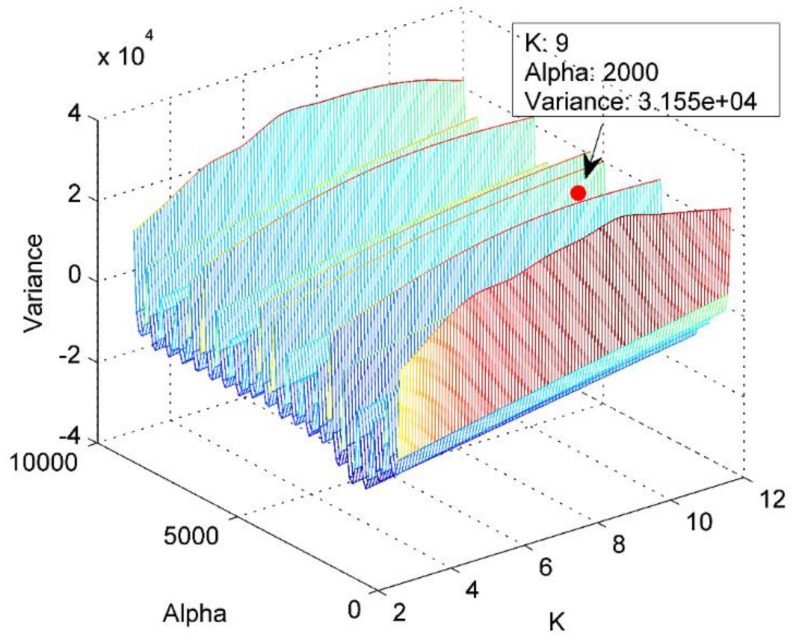
The variance with respect to the combination of *K* and α.

After that, the mode number *K* is set to 3–12, and the quadratic penalty term α is set as 500–10000 with step size of 500. The VMD is then performed on the simulation signals with the variance of the IMFs’ center frequency after each decomposition calculated. The paired parameters maximizing the variance are exactly the optimal parameters for IVMD. Figure 3 shows the variance with respect to the combination of *K* and α.

**Figure 4:**
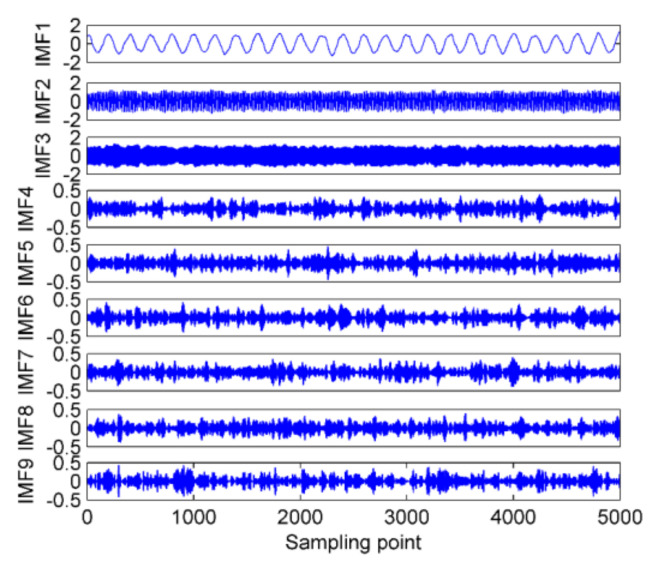
The IVMD results of the simulation signals.

As shown in Figure 3, the combination of mode number and quadratic penalty term maximizing the variance is (9,2000), hence, K=9 and α=2000 are considered as the optimal parameters for IVMD. In this case, it means a significant overall difference in the IMFs’ center frequency. The decomposition results of the simulation signals using IVMD with the obtained optimal parameters is shown in Figure 4.

Table 1 lists the distribution of the IMFs’ center frequency by the proposed IVMD method. It can be noted from Table 1 that the three components corresponding to the simulation signals can be deduced correctly. The above simulation experiments have reasonably confirmed the feasibility and effectiveness of using the variance of IMFs’ center frequency to lock the mode number and quadratic penalty term for IVMD. In addition, according to the decomposition result, we can also noticed that *K* acts as a decisive factor influencing the decomposition result, while the influence exerted by α on the result is relatively small.

In order to further highlight the decomposition advantages of IVMD, the correlation coefficients (CCs) between IMFs and simulation signals for EMD, EEMD, and IVMD are calculated. The results are listed in Table 2. As shown in Table 2, the CCs between the corresponding IMFs by IVMD and the raw simulation signals are obviously larger than that by the other methods. Hence, IVMD can accurately dig out the single frequency components hidden in the composite signals with the best decomposition performance.

### 4.2. Denoising of Simulation Signals

Combined with the analysis in Section 4.1, it can be concluded that among the IMFs obtained by the noisy signals processed by the signal decomposition algorithms, not every IMF can characterize the original signals.

**Figure 5:**
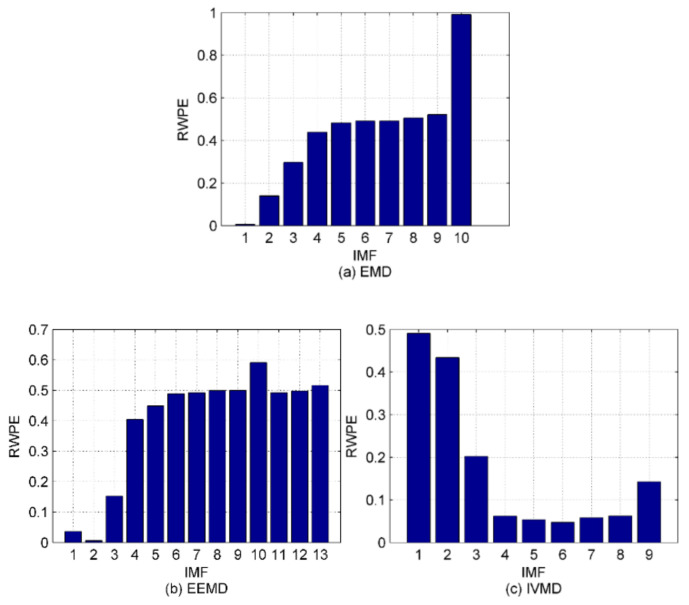
The histogram of the RWPE results for EMD, EEMD and IVMD; (**a**) EMD method; (**b**) EEMD method; (**c**) IVMD method.

Some components are noise and they should be excluded before feature extraction. Therefore, the simulation signals in Section 4.1 are again introduced to test the performance of the proposed denoising method (described in Section 3). First, we calculate the RWPE values of IMFs by EMD, EEMD and IVMD. As suggested in [15], the time delay and embedding dimension are set as one and five, respectively. The histogram of the calculation results is shown in Figure 5.

We can easily observe from Figure 5 that the noise IMFs and the remaining ones can be clearly distinguished for EMD and EEMD, when the threshold of RWPE is set to 0.1. As for the IVMD method, almost all the noise IMFs have been filtered out except IMF_9_. Intuitively, the denoising method is feasible to suppress the in-band noise hidden in the raw SRN signals. To further validate the effectiveness of the method, the IMFs with RWPE values greater than 0.1 are selected to reconstruct new signals. Table 3 lists the SNR and root mean square errors (RMSE) of the noisy simulation signal and the reconstructed signals under different algorithms. Table 3 shows that after the noisy signal processed by IVMD-RWPE, both SNR and RMSE have been significantly improved with better performance than EMD-RWPE and EEMD-RWPE. Hence, the proposed IVMD-RWPE method is more powerful for denoising thanks to its effectiveness and reliability.

### 4.3. Analysis of PE Properties

**Figure 6:**
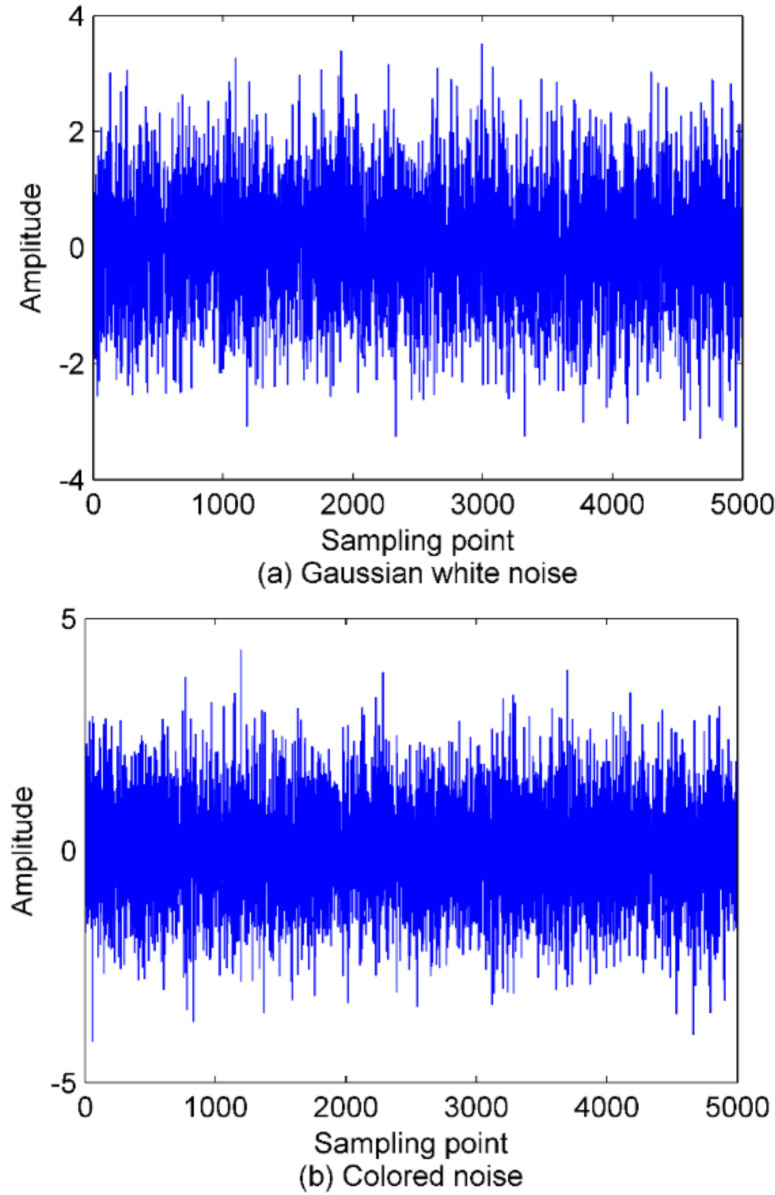
The time-domain waveforms for Gaussian white noise and colored noise. (**a**) the Gaussian white noise series; (**b**) the colored noise series.

In order to explore the effect of data length on PE, the standard Gaussian white noise and colored noise series with length of 5000 are constructed, respectively. Then we calculate the PE, AE, and SE of the noise with a step size of 100. In this way, each entropy has a total of 50 values. Figure 6 shows the time-domain waveforms of the two types of noise.

**Figure 7:**
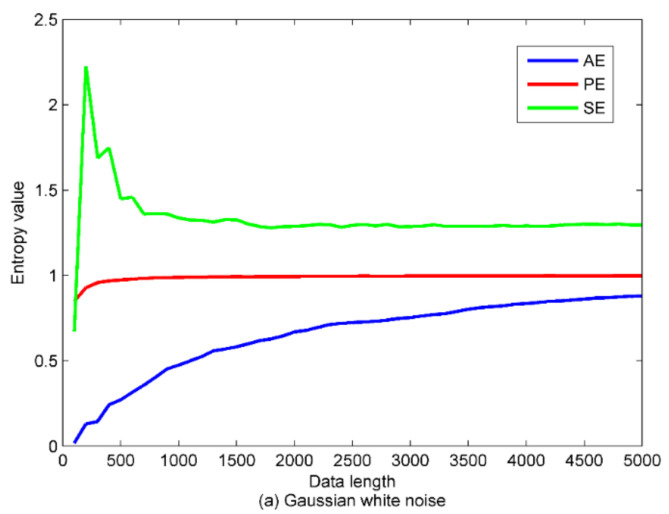

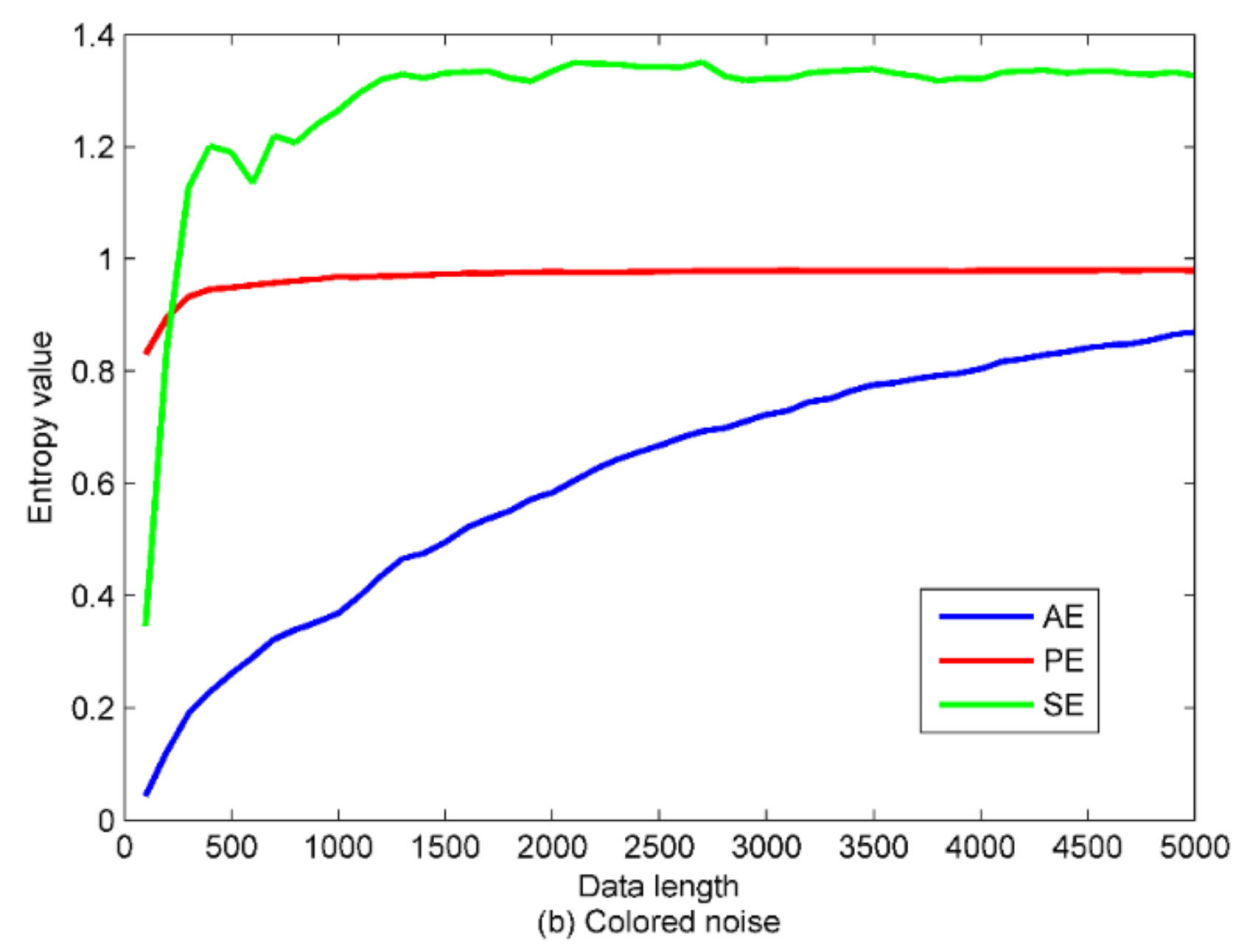
The calculation results of AE, PE and SE for Gaussian white noise and colored noise. (**a**) the Gaussian white noise series; (**b**) the colored noise series.

The calculation results of entropy are presented in Figure 7. It can be observed from Figure 7, as the data length increases, the PE value first increases and then converges to a fixed value. By contrast, the trend of SE is swinging more wildly, also, the AE value increases with the data length increasing without convergence. In summary, PE is more desirable in the application of real-time feature extraction due to its being stable and computationally fast. Based on the above analysis, the data length in this paper is uniformly set to 5000.

## 5. Feature Extraction of SRN Signals Based on IVMD-norMIC-PE

### 5.1. IVMD of SRN Signals

**Figure 8:**
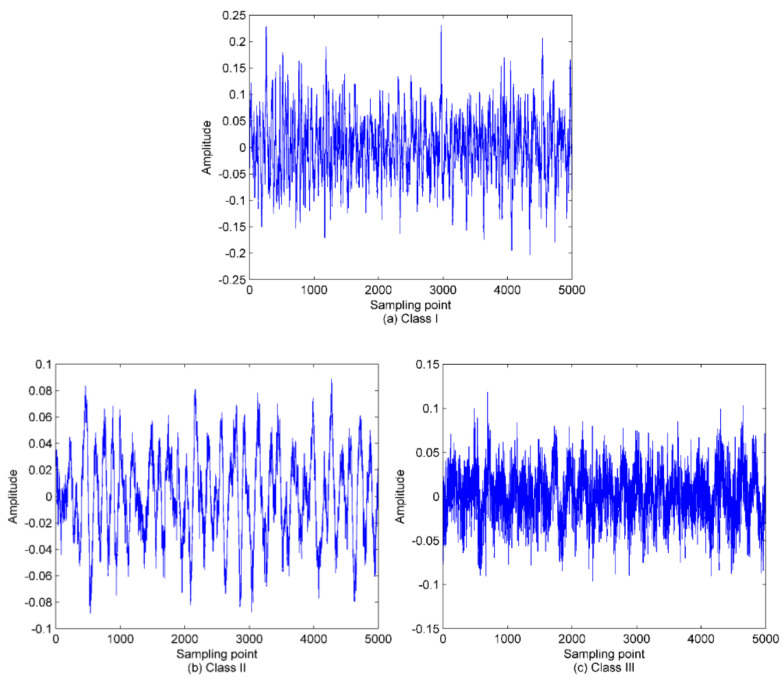
The time-domain waveforms of normalized SRN signals. (**a**) Class I; (**b)** Class II; (**c**) Class III.

**Figure 9:**
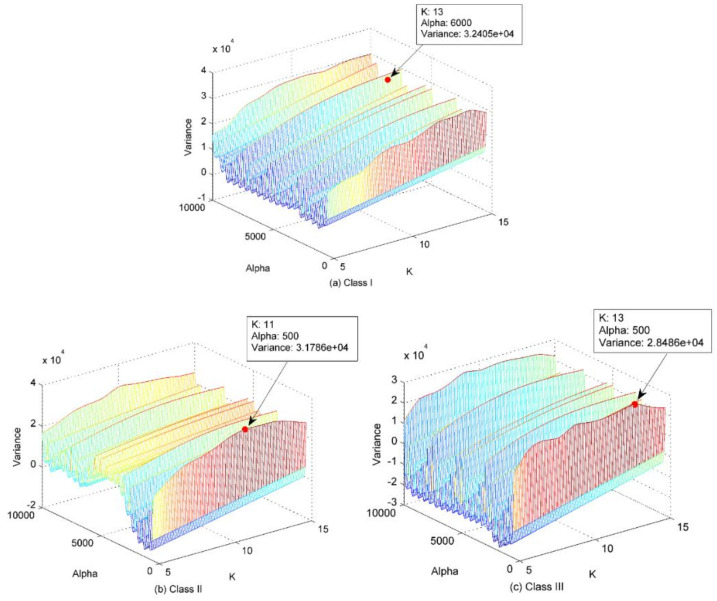
The variance with respect to the combination of *K* and α for the three types of SRN signals. (**a**) Class I; (**b**) Class II; (**c**) Class III.

**Figure 10:**
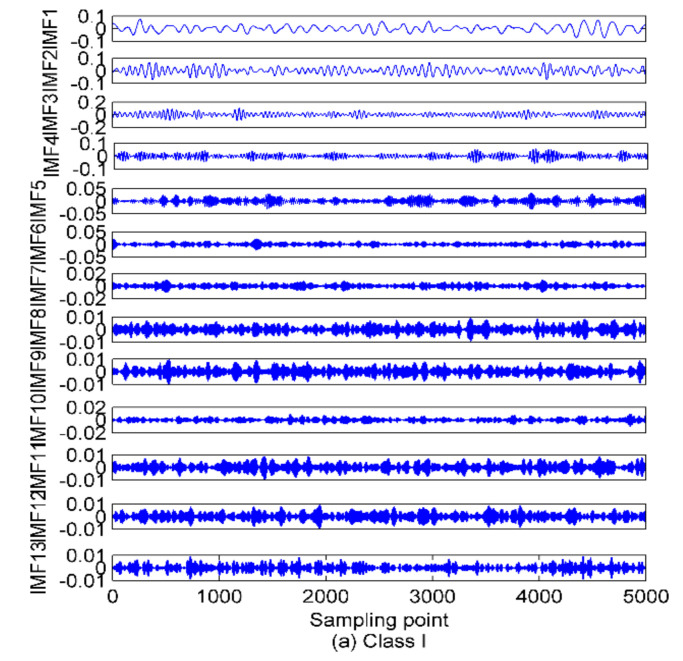

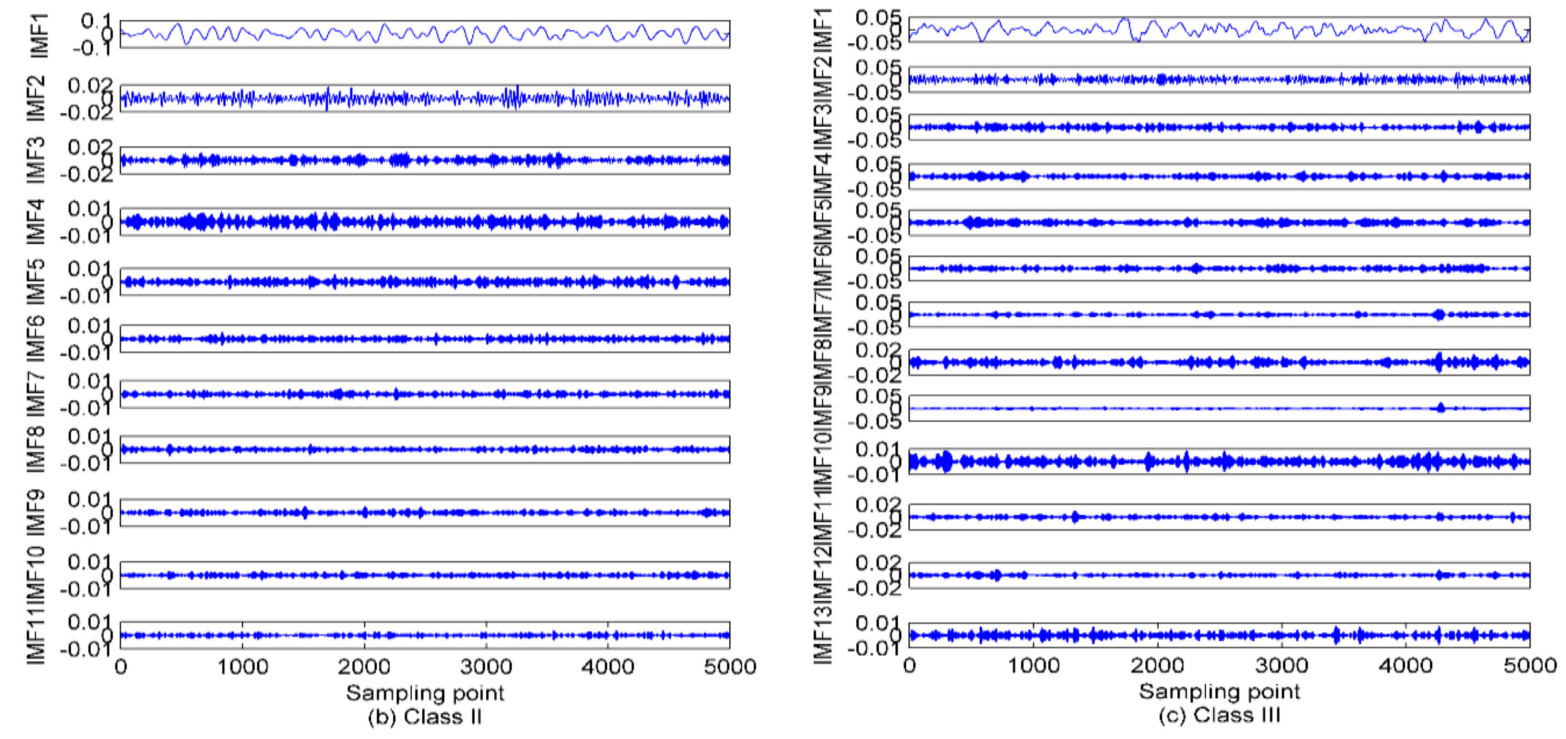
The IVMD results of the SRN signals. (**a**) Class I signal; (**b**) Class II signal; (**c**) Class III signal.

In this paper, three types of SRN signals are randomly selected from a real measured data set in [36], namely, Class I, Class II and Class III. One hundred samples are randomly selected from each category, thus, a total of 300 SRN samples are used for the analysis. The sampling frequency is 52.734 kHz, and for other measurement parameters, please refer to [36] for more details. The time-domain waveforms of the normalized SNR signals are shown in Figure 8. As presented in the proposed method, the mode number *K* and quadratic penalty term α should be calculated before IVMD of SRN signals. The variance with respect to the combination of *K* and α for the three types of SRN signals are given in Figure 9. As shown in Figure 9, the optimal combination of parameters for the three types of signals are $\left(13,6000\right)$, $\left(11,500\right)$ and $\left(13,500\right)$, respectively. Figure 10 is the IVMD results of the SRN signals.

### 5.2. Denoising of SRN Signals

**Figure 11:**
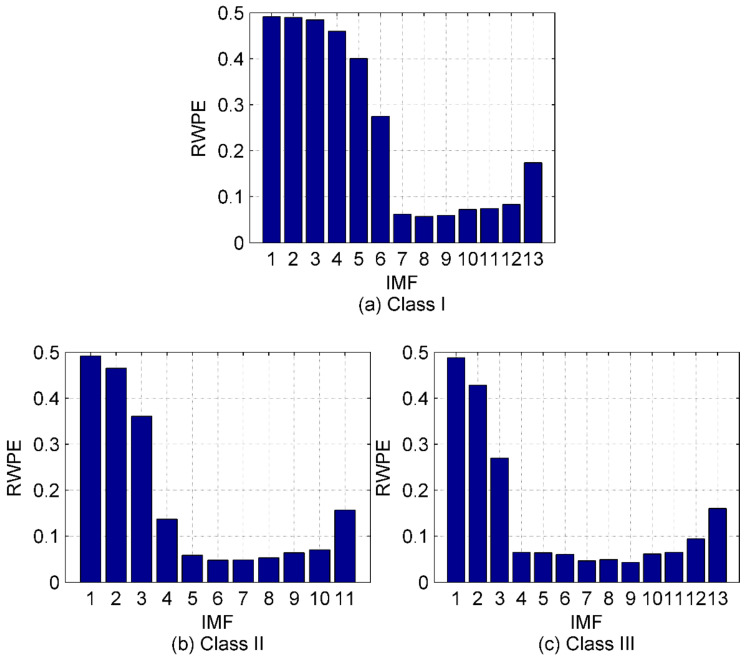
The RWPE distribution of IMFs by IVMD for the three types of SRN signals; (**a**) Class I signal; (**b**) Class II signal; (**c**) Class III signal.

Figure 8 shows that owing to the pollution of marine environmental noise, the time-domain waveforms of the raw SRN signals become unclear. In this way, the proposed denoising method is desirable in this case for further processing of the noisy signals. Figure 11 is the RWPE distribution of IMFs by IVMD for the three types of SRN signals. The IMFs with RWPE value greater than 0.1 are then selected for the subsequent feature extraction of SRN signals. One thing that needs to be noted here is that the residual components by EMD and EEMD should be excluded.

### 5.3. Classification of SRN Samples

**Figure 12:**
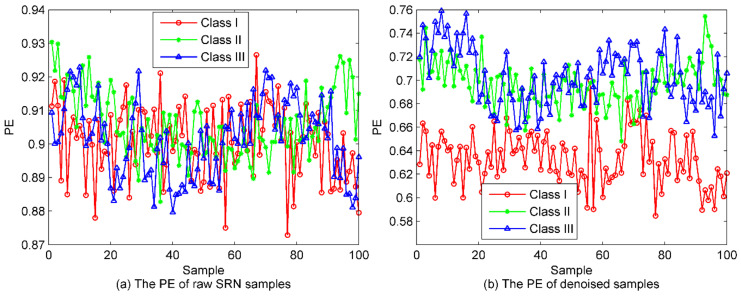
The PE for the raw SRN samples and the denoised ones; (**a**) The PE of raw SRN samples; (**b**) The PE of denoised samples.

**Figure 13:**
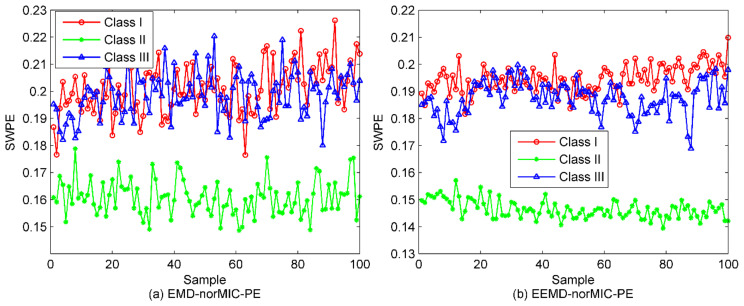

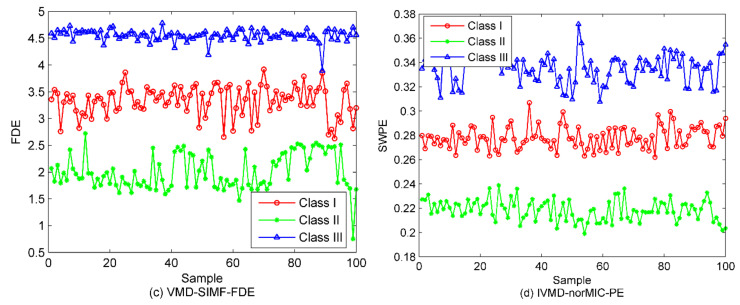
The entropy distribution under different algorithms. (**a**) EMD-norMIC-PE; (**b**) EEMD-norMIC-PE; (**c**) VMD-SIMF-FDE; (**d**) IVMD-norMIC-PE.

To demonstrate the necessity of the denoising method, fist, the selected IMFs in Section 5.2 are utilized for the reconstruction of new SRN signal. Then, we can easily calculate the PE of the raw SRN samples and the denoised ones. The results are presented in Figure 12. As seen from Figure 12, as a result of marine environmental noise, the three kinds of SRN samples are mixed together and cannot be recognized at all. In contrast, after denoising of the SRN signals, a large proportion of the samples in Class I have been separated, while the samples of Classes II and III still remain unrecognizable. Besides, it is worth noting that compared with the raw SRN samples, the PE values of the denoised samples start to decrease, further confirming the validity of the proposed denoising algorithm. For fair comparison, the entropy distribution of EMD-norMIC-PE, EEMD-norMIC-PE, VMD-SIMF-FDE [31] and IVMD-norMIC-PE are shown in Figure 13.

As seen from Figure 13, for EMD-norMIC-PE, Class II samples are separated from the mixed samples, the performance has been greatly improved compared to the PE-based method. As far as EEMD-norMIC-PE is concerned, in addition to the identifiable Class II samples, some samples of Class I and Class III have also begun to separate. In contrast, the three types of samples can be clearly distinguished by the VMD-SIMF-FDE method with a small number of cross samples. By contrast, the proposed IVMD-norMIC-PE method performs best in terms of the separation degree of these samples with a significantly larger inter-class spacing.

After this, 60 randomly selected samples from each class are used to train the PSO-SVM multi-class classifier, and the remaining 40 ones are used as the test data. The MATLAB codes of PSO-SVM multi-class classifier can be downloaded from [37]. The classification outputs of the classifier are shown in Table 4. As illustrated in Table 4, the largest number of misclassified samples appear in the PE-based method with a recognition rate of only 36.6667%, which is far from the standard in practical applications. By comparison, the classification accuracy of the proposed IVMD-norMIC-PE technique is up to 99.1667%, which is much higher than that of other schemes. The experimental results manifest what the incomparable superiority the proposed method has compared with other currently existing methods.

## 6. Conclusions

In order to extract the inherent features that can characterize ship-radiated noise, a technique fully integrating IVMD, norMIC and PE is presented in this paper. The IVMD addressed the mode number, as well as the quadratic penalty term issue for VMD. Thus, it is employed to decompose the SRN signals for the first time. As a kind of correlation metric, MIC is more accurate than MI in measuring the correlation between paired variables. PE enjoys the merits of being conceptually simple and computationally fast, and has better stability than AE and SE. The proposed technique inherits the advantages of IVMD, MIC coupled with PE in a proper manner. In stark contrast to other methods, the proposed technique employs RWPE to screen sensitive IMFs, thereby paving the way for the subsequent PE extraction. Furthermore, the norMICs are used to weigh the PE results according to the degree of correlation between the sensitive IMFs and the raw SRN signal. The excellent performance of the proposed method has been reasonably verified by the classification experiment of ships. The experimental results reveal that the proposed method has a 99.1667% classification accuracy, which is much higher than other schemes. On this basis, the proposed technique is more reliable in terms of feature extraction and classification in practice. In future work, we will try to explore other parameter selection methods for VMD and denoising algorithms to further improve the performance of the system.

## Figures and Tables

**Table 1:** The distribution of IMFs’ center frequency by improved variational mode decomposition (IVMD).

Methods	Center Frequency/Hz								
IVMD	5.09	50.02	100.02	184.55	246.22	307.54	364.42	417.07	471.72

**Table 2:** The distribution of correlation coefficients between IMFs and simulation signals.

	EMD	EEMD	IVMD
*f*_1_	IMF_6_: 0.9156	IMF_7_: 0.9769	IMF_1_: 0.9899
*f*_2_	IMF_3_: 0.8263	IMF_4_: 0.9383	IMF_2_: 0.9895
*f*_3_	IMF_2_: 0.8280	IMF_3_: 0.9078	IMF_3_: 0.9866

**Table 3:** The signal to noise ratio (SNR) and RMSE under different algorithms.

	Before Denoising	EMD-RWPE	EEMD-RWPE	IVMD-RWPE
SNR/db	7.6921	10.8008	12.0176	15.8777
RMSE	0.5052	0.3532	0.3070	0.2387

**Table 4:** The classification results under different algorithms.

Method	Number of Misclassified Samples			Recognition Rate (%)
	Class I	Class II	Class III	
PE before denoising	24	20	32	36.6667
PE after denoising	2	11	30	65.8333
EMD-norMIC-PE	26	0	7	72.5
EEMD-norMIC-PE	7	0	17	80
VMD-SIMF-FDE (31)	2	0	1	97.5
IVMD-norMIC-PE	1	0	0	99.1667
